# PPIC, EMP3 and CHI3L1 Are Novel Prognostic Markers for High Grade Glioma

**DOI:** 10.3390/ijms17111808

**Published:** 2016-10-28

**Authors:** Yuan-Feng Gao, Tao Zhu, Chen-Xue Mao, Zhi-Xiong Liu, Zhi-Bin Wang, Xiao-Yuan Mao, Ling Li, Ji-Ye Yin, Hong-Hao Zhou, Zhao-Qian Liu

**Affiliations:** 1 Department of Clinical Pharmacology, Xiangya Hospital, Central South University, Changsha 410008, China; gaoyuanfeng126@126.com (Y.-F.G.); euzhutao@hotmail.com (T.Z.); sharemix@163.com (C.-X.M.); wangzhibinwalking@163.com (Z.-B.W.); xiaoyuanm@csu.edu.cn (X.-Y.M.); yx132liling@126.com (L.L.); yinjiye@csu.edu.cn (J.-Y.Y.); HHzhou2003@163.com (H.-H.Z.); 2 Institute of Clinical Pharmacology, Central South University, Hunan Key Laboratory of Pharmacogenetics, Changsha 410078, China; 3 Department of Neurosurgery, Xiangya Hospital, Central South University, Changsha 410008, China; zhixiongliu@csu.edu.cn

**Keywords:** novel glioma markers, DEPGs, *PPIC*, *EMP3*, *CHI3L1*

## Abstract

Current treatment methods for patients diagnosed with gliomas have shown limited success. This is partly due to the lack of prognostic genes available to accurately predict disease outcomes. The aim of this study was to investigate novel prognostic genes based on the molecular profile of tumor samples and their correlation with clinical parameters. In the current study, microarray data (GSE4412 and GSE7696) downloaded from Gene Expression Omnibus were used to identify differentially expressed prognostic genes (DEPGs) by significant analysis of microarray (SAM) between long-term survivors (>2 years) and short-term survivors (≤2 years). DEPGs generated from these two datasets were intersected to obtain a list of common DEPGs. The expression of a subset of common DEPGs was then independently validated by real-time reverse transcription quantitative PCR (qPCR). Survival value of the common DEPGs was validated using known survival data from the GSE4412 and TCGA dataset. After intersecting DEPGs generated from the above two datasets, three genes were identified which may potentially be used to determine glioma patient prognosis. Independent validation with glioma patients tissue (*n* = 70) and normal brain tissue (*n* = 19) found *PPIC*, *EMP3* and *CHI3L1* were up-regulated in glioma tissue. Survival value validation showed that the three genes correlated with patient survival by Kaplan-Meir analysis, including grades, age and therapy.

## 1. Introduction

As the most common primary central nervous system malignancy in humans, glioma is characterized by the existence of heterogeneous cells that are involved in disease progression [1]. Generally speaking, gliomas are divided into two classes: low-grade gliomas (LGG, I or II) that display relatively slow growth, and high-grade gliomas (HGG, III or IV) characterized by rapid growth and invasion into normal brain tissue [2]. Gliomas are highly fatal and the majority of HGG patients suffer from a poor quality of life [3,4]. Median survival time of patients with glioblastoma multiforme (GBM)—the most common and most malignant type of glioma—is only 14.6 months, and the five-year survival rate is less than 10% [5,6]. Increasing efforts are made to improve HGG treatment. Recently, several studies have focused on using gene expression profiles to identify potential new biomarkers for diagnosis, prognosis, staging and therapy development [7,8]. Multiple studies suggest that gene expression-based classification of malignant gliomas may correlate better with survival than histological classification [9], and provide a useful method to identify previously unrecognized but clinically relevant prognostic indicators [10,11]. To date, microarray analysis has been successfully used to identify potential glioma-related genes [12] and analyze gene expression levels within certain biological networks [13,14,15]. However, evidence from microarray analysis of gliomas suggests that examination of a single gene offers limited information with poor clinical outcome correlations [16,17].

Taken collectively, the failure to empirically develop an effective treatment for glioma emphasizes the need to utilize the molecular profile of tumor samples and its correlation with clinical parameters to develop rationally designed treatment strategies. In this study, we identified three differentially expressed prognostic genes (DEPGs) that were common among two glioma expression profiles. Gene expression was validated by real-time quantitative reverse transcription PCR (qPCR). We determined that *PPIC*, *EMP3* and *CHI3L1* were up-regulated in the glioma tissue. The survival value of the DEPGs was validated using known survival data from the expression profiles and TCGA datasets. Kaplan-Meir analysis of the datasets revealed that the three genes were correlated with survival, including grades, age and therapy. Taken together, the results suggest that the genes may be suitable biomarkers for diagnostic or therapeutic strategies for high-grade gliomas.

## 2. Results

### 2.1. Identification of DEPGs

**Figure 1:**
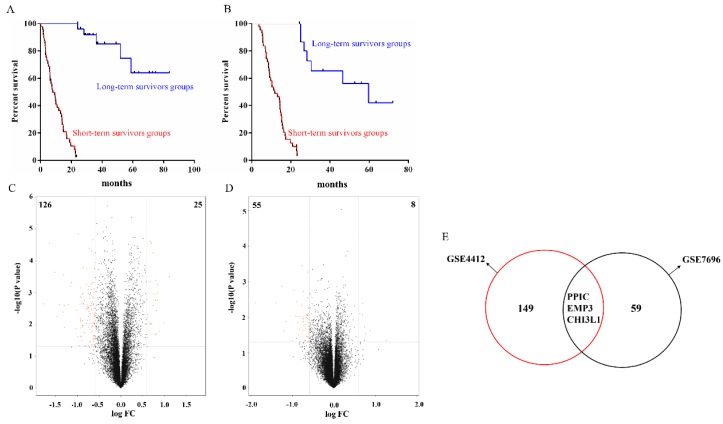
Identification of DEPGs. (**A**,**B**) There was significant difference between long-term and short-term survivors groups both in GSE4412 and GSE7696 datasets by Kaplan-Meier; (**C**) 151 genes between long-term survivors and short-term survivors in GSE4412 were filtered as DEPGs, including 25 up-regulated and 126 down-regulated genes; (**D**) A total of 63 genes between normal and tumor tissues in GSE7696 were filtered as DEPGs, including 8 up-regulated and 55 down-regulated genes; (**E**) After intersection, a total of three common DEPGs were detected.

Two gene expression profiles (GSE4412 and GSE7696) of long-term survivors’ (>2 years) and short-term survivors’ (≤2 years) glioma tissue samples were analyzed to identify genes differentially expressed. As shown in Figure 1A,B, there was significant difference between long-term and short-term survivors groups both in GSE4412 and GSE7696 datasets by Kaplan-Meier analysis (*p* < 0.001 and *p* < 0.001, respectively). A total of 151 genes (25 up-regulated and 126 down-regulated genes) in GSE4412 and 63 genes (8 up-regulated and 55 down-regulated genes) in GSE7696 were identified as differentially expressed prognostic genes (DEPGs) between long-term survivors and short-term survivors (Figure 1C,D, Table S1). Intersection of the DEPGs revealed a total of three common DEPGs—*PPIC*, *EMP3* and *CHI3L1*—suggesting that these common DEPGs may be potential prognostic indicators of glioma progression (Figure 1E). 

### 2.2. Independent Validation of Glioma-Specific Markers

**Figure 2:**
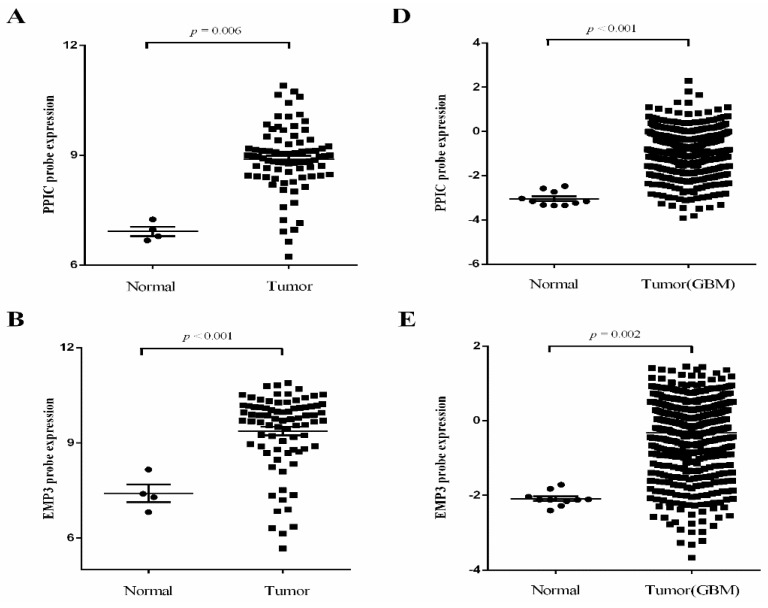

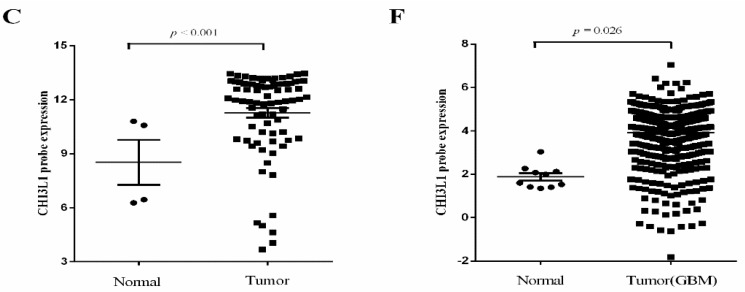
Independent validation of glioma-specific markers in GSE7696 and The Cancer Genome Atlas (TCGA). (**A**–**C**) Analysis of the GSE7696 data revealed that *PPIC*, *EMP3* and *CHI3L1* have >2-fold up-regulation at the transcription level and were drastically increased in malignant gliomas when compared to non-tumor brain tissue; (**D**–**F**) The expression changes of *PPIC*, *EMP3* and *CHI3L1* are consistent with those in the TCGA datasets.

We further validated the three relevant prognostic genes by expression profile and real-time reverse transcription quantitative PCR (qPCR). We analyzed expression of *PPIC*, *EMP3* and *CHI3L1* in expression profiles acquired from the Gene Expression Omnibus (GEO) database, and glioma datasets acquired from The Cancer Genome Atlas (TCGA). Analysis of the GSE7696 data revealed that *PPIC*, *EMP3* and *CHI3L1* have greater than two-fold up-regulation at the transcription level and were drastically increased in malignant gliomas when compared to non-tumor brain tissue (Figure 2A–C). It is also noteworthy that expression changes of these genes are consistent with those in the TCGA datasets (Figure 2D–E).

**Figure 3:**
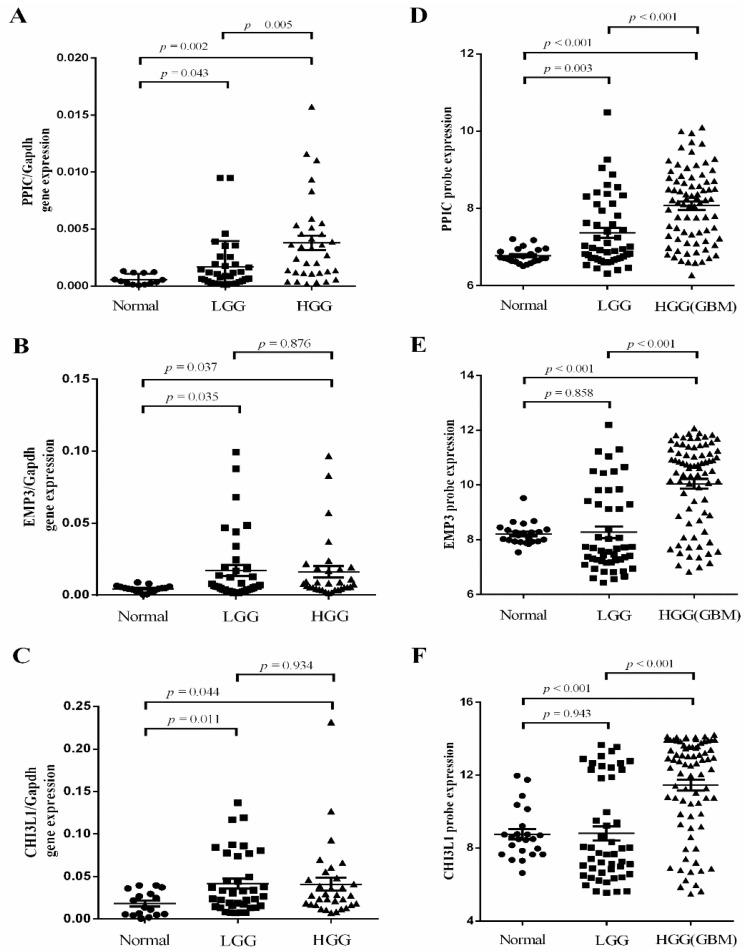
Independent validation of glioma-specific markers. Three related genes were validated by real-time quantitative reverse transcription-PCR. (**A**) The expression of *PPIC* expression was significantly higher in malignant gliomas compared to lower grade gliomas and non-tumor brain tissue. *PPIC* expression directly correlated with glioma grade; (**B**,**C**) *EMP3* and *CHI3L1* expression was drastically increased in malignant gliomas, but no directly correlated with the glioma grade; (**D**–**F**) *PPIC*, *EMP3* and *CHI3L1* expression was drastically increased between gliomas and normal tissue in GSE4290 data.

To further validate these results, expression of the three genes was validated by real-time quantitative reverse transcription-PCR (qPCR). We found that expression of *PPIC* expression was significantly higher in malignant gliomas compared to lower grade gliomas and non-tumor brain tissue. *PPIC* expression directly correlated with glioma grade (Figure 3A), and was upregulated more than two-fold as determined by GSE4290 (Figure 3D). Additionally, our results showed that *EMP3* and *CHI3L1* expression was significantly higher in gliomas compared to non-tumor brain tissue. No correlations between gene expression and glioma grade were identified (Figure 3). Furthermore, the associations between DEPGs expression and clinicopathological parameters were analyzed in the present study. No significant association was observed between target gene expression and patient age or gender (Table 1). Taken together, the results suggest that expressions of *PPIC*, *EMP3* and *CHI3L1* may play a vital role in glioma progression.

### 2.3. Survival Value Validation of Patients with Grades III and IV Gliomas by the Three-Gene Signature

**Figure 4:**
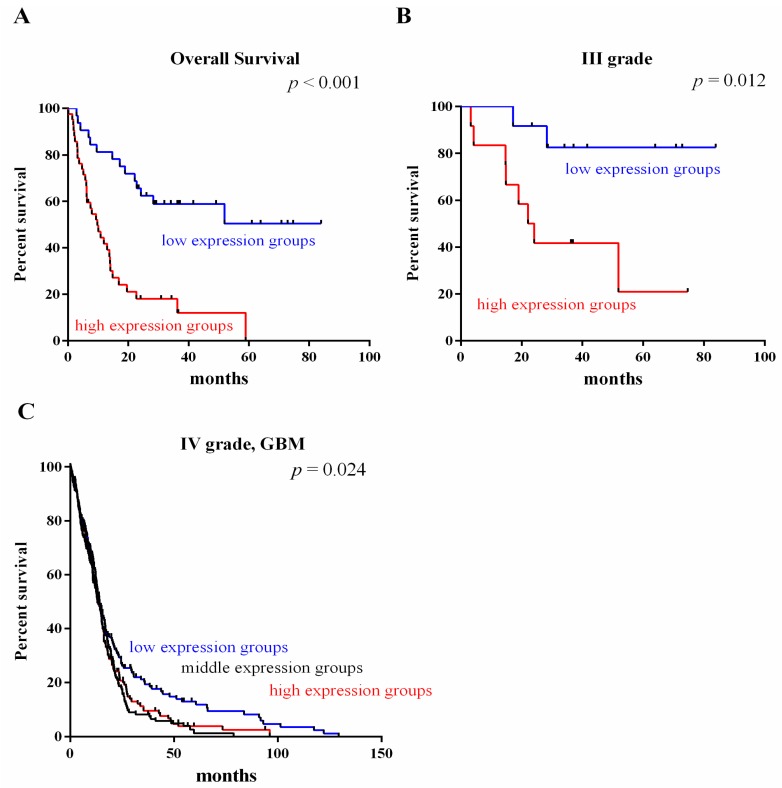
Survival value validation of patients with grades III and IV gliomas by the three-gene signature. (**A**,**B**) The three-gene signature classified patients into low mRNA and high mRNA expression groups which differed in overall survival and grade III of GSE4412 dataset significantly; (**C**) The three-gene signature significantly separated patients in IV (GBM) into high and low expression groups in TCGA dataset. The *p*-values were computed by the log-rank test.

To investigate the relationship between the expression of the validated DEPGs and patients’ survival, we analyzed the prognostic significance of the genes using Kaplan-Meier analysis for expression profile GSE4412 and TCGA datasets. The three-gene signature classified patients into low mRNA and high mRNA expression groups, which differed in overall survival of HGG significantly, reflecting the biological characteristics and heterogeneity of the glioma grade (GSE4412, Figure 4A). In Kaplan-Meier analysis, the three-gene signature significantly separated patients in grade III (GSE4412, Figure 4B) and IV (GBM TCGA, Figure 4C) into high and low expression groups. These results indicated that low expression of the three-gene signature probably confers a survival advantage to glioma patients. These results collectively suggested the prognostic value of the three-gene signature expression. 

### 2.4. Survival Value Validation of Patients with Age by the Three-Gene Signature

**Figure 5:**
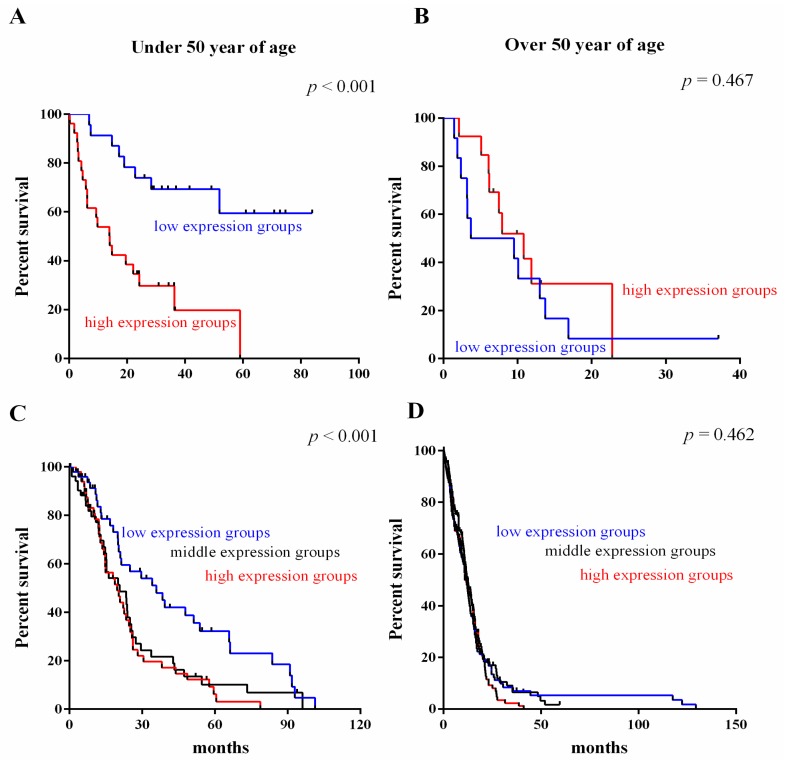
Survival value validation of patients with age by the three-gene signature. Patients were classified into groups of people under 50 (young patients) and over 50 (old patients) years of age. In young patients groups, the three-gene signature significantly stratified patients set into high and low expression groups both in GSE4412 (**A**) and TCGA(**C**); However, it could not significantly classify in old patients both in GSE4412 (**B**) and TCGA (**D**). The *p*-values were computed by the log-rank test.

To investigate the association of the three-gene signature with age, patients were classified into groups of people under 50 (young patients) and over 50 (old patients) years of age. Diagnosis with gliomas at a younger age (under 50) is a strong predictor of longer patient survival. As shown in Figure 5, it could not significantly classify in old patients (Figure 5B,D) both in GSE4412 and TCGA. However, in young patients groups, the three-gene signature significantly stratified patients set into high and low expression groups (Figure 5A,C). Consistent with recent reports showing that patients under 50 years of age have more favorable prognosis than patients over 50 years old our study classified most patients under 50 years as low risk and over 50 years of age as high risk.

### 2.5. Survival Value Validation of Patients with Temozolomide (TMZ) and Radiotherapy by the Three-Gene Signature

**Figure 6:**
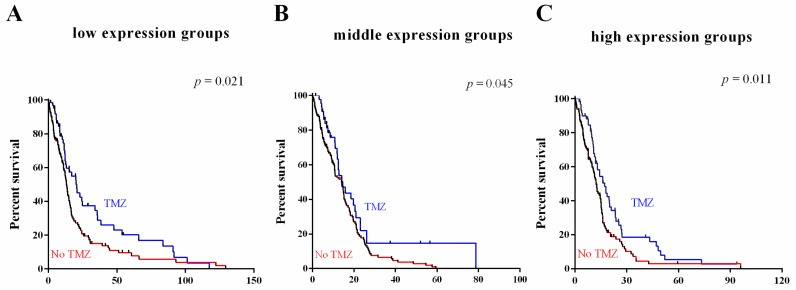

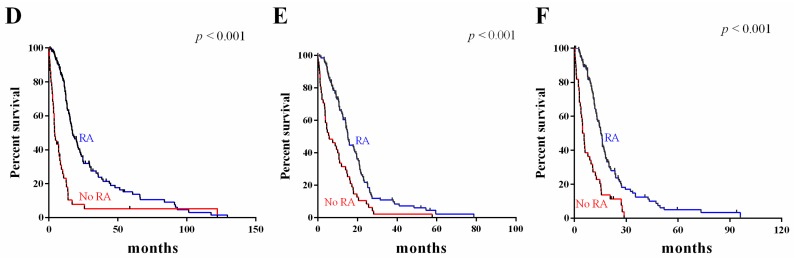
Survival value validation of patients with TMZ and radiotherapy by the three-gene signature. (**A**–**C**) Patients in low, middle and high expression groups with TMZ in TCGA dataset; (**D**,**E**) Patients in low, middle and high expression groups with radiotherapy in TCGA data set. The *p*-values were computed by the log-rank test.

To determine the association of the three-gene signature with response to chemotherapy and radiotherapy, subset analyses were performed on TCGA dataset, for which therapeutic information was available. As shown in Figure 6, patients in different groups benefitted from TMZ therapy (Figure 6A–C) and radiation therapy (Figure 6D–E). These results indicated that expression of the three-gene signature is indicative of a positive response to adjuvant chemotherapy and radiotherapy.

## 3. Discussion

Recently, microarray-based expression profiling studies have revealed molecular events of glioma which could be of prognostic value [18]. Studies based on gene expression datasets have been reported to classify patients according to known prognostic factors. Unfortunately, no report has yet predicted chemotherapy response in gliomas. In this study, microarray data from GSE4412 and GSE7696 datasets were used to identify differentially expressed prognostic genes (DEPGs) between long-term and short-term survivors. After intersecting DEPGs generated from the above two datasets, three common DEPGs (*PPIC*, *EMP3* and *CHI3L1*) were identified, suggesting that these genes may be potential predictors of prognosis in high-grade gliomas patients. Besides, our qPCR results suggest that expressions of *PPIC*, *EMP3* and *CHI3L1* may provide new biomarkers to assist in clinical decision-making concerning new opportunities for targeted treatment of individual patients. 

Among them, *PPIC* encodes Cyclophilin C (*Cyp-C*), an enzyme that is part of the cyclophilin family. Human Cyclophilin C was isolated from a human kidney cDNA library [19]. Researchers found that cyclophilins have been implicated in the folding and function of multiple proteins in various cellular compartments [20,21,22]. Cyclophilin C-associated protein (*CyCAP*) is a 77 kDa intracellular protein, with a scavenger-receptor cysteine-rich domain that is released into the cytoplasm and cell membrane [23,24,25]. The expression of *CyCAP* has been shown to be elevated by wounds and ischemia due to the presence of interferon-gamma (IFNγ) and tumor necrosis factor-alpha (*TNFα*) [26,27]. In addition, the deletion of *CyCAP* abolished a 45 kDa fibronectin-induced *MMP-13* expression [28,29]. However, the reports about *PPIC* and glioma were very limited and the detailed mechanism in glioma is still unclear. 

Another potential predictor of glioma prognosis is *EMP3*. *EMP3* is a member of the peripheral myelin protein 22-kDa (*PMP22*) gene family (also known as the *TMP* gene family). The *PMP22* gene family is comprised of five members including *PMP22*, *EMP3*, *EMP2*, and *EMP1*, and *MP20* [30,31]. The *EMP3* gene encodes protein epithelial membrane protein 3 (*EMP-3*), a 163-amino acid protein containing four transmembrane domains and two N-linked glycosylation sites in the first extracellular loop. *EMP3* is thought to participate in cell proliferation and cell-cell interactions [30,31]. A study by *Alaminos M* showed that *EMP3* reintroduction in *EMP3*-deficient cancer cells inhibits colony formation and tumor growth in xenografts, indicating a tumor suppressing function of the *EMP3* gene. *EMP3* has also been reported to show frequent promoter methylation in high-grade astrocytomas and neuroblastomas [32], two tumor entities that display frequent allelic deletions at 19q13.3 [33,34,35]. 

Additionally, *CHI3L1*, also known as *YKL-40*, is a secreted 40 kDa glycoprotein that is upregulated in several human cancers and other diseases characterized by chronic inflammation [36]. A wealth of clinical evidence has revealed that elevated serum levels of *CHI3L1* in GBM are positively correlated with cancer invasiveness, radioresistance, recurrence, and reduced patient survival times [37,38]. There are data that suggest that *CHI3L1* expression could be a prognostic predictor of glioblastoma [39,40,41]. This is the same as what we found. 

Of interest was the fact that our univariate analysis of patients with available TMZ information suggested that the three-gene signature could predict patients who would benefit from TMZ. The utility of the gene signature for treatment management in glioma still needs to be further evaluated in a prospective TMZ clinical trial. In addition, the three-gene signature has the ability to identify patients benefiting from radiotherapy. Therefore, the established gene signature might be helpful in clinical management.

Reasonable use of microarray datasets not only allows for quicker and simpler analysis of large quantities of biological information, but also facilitates the accurate identification of key molecular mechanisms. In the present study, we provided the expression of these potential markers, *PPIC*, *EMP3* and *CHI3L1*. We also identified and validated the prognostic value of novel candidate genes. However, their role in glioma development is largely unknown. Therefore, further studies are required to more precisely characterize the functional significance of these genes in glioma progression, and their potential application for glioma prognosis.

## 4. Materials and Methods

### 4.1. Affymetrix Microarray Data

Expression profiles (GSE4412, GSE7696 and GSE4290) were acquired from the Gene Expression Omnibus (GEO, http://www.ncbi.nlm.nih.gov/geo/) database. The platform of GSE4412 is GPL96 [HG-U133A] Affymetrix Human Genome U133A Array. The platform of GSE7696 and GSE4290 is GPL570 [HG-U133_Plus_2] Affymetrix Human Genome U133 Plus 2.0 Array. Only grade III (*n* = 24) and IV (*n* = 50) gliomas were included in GSE4412. Sixty glioblastoma multiforme (GBM) were included in GSE7696. In GSE4290 data, 157 glioma samples, including astrocytoma, oligodendroglioma and glioblastoma samples (45 grade II, 31 grade III and 81 grade IV) were used. The original CEL files as well as the probe annotation were downloaded from the platform. Moreover, we used public TCGA (http://cancergenome.nih.gov/) data repositories as our other source of samples, and a total of 567 tumors having clinical data were profiled for class discovery and survival analysis. Detailed information used for these datasets are described in Table 2.

### 4.2. Differentially Expressed Prognostic Genes (DEPGs) Analysis

The probe-level data in CEL files were converted into expression profiles and the robust multiarray average (RMA) with affy package was used to correct the background and normalize quartile data. For genes with multiple corresponding probe sets, which have a plurality of expression values, the gene expression values reflect the averaged values of those probe sets [42,43]. All the patients were classified into long-term survivors (>2 years) and short-term survivors (≤2 years). Genes were considered differentially expressed when meeting the cut-off criterion of |log fold change (FC)| ≥ 1.5 and *p* < 0.05. The results generated from GSE4412 and GSE7696 datasets were then intersected to find DEPGs. These genes were further validated utilizing GSE4412 and TCGA datasets with known survival data to determine their prognostic significance in GBM patients.

### 4.3. Patients and Tissue Samples

Seventy patients with grade I to IV gliomas that underwent surgical resection in Hunan Cancer Hospital (Changsha, China) between 2007 and 2013 were enrolled in this institutional review board-approved study. Nineteen normal brain samples (from patients with brain injuries) were collected for controls. Tumors were histopathologically classified according to the WHO classification. The tissue samples were flash frozen in liquid nitrogen immediately after resection and stored at −80 °C until further processing.

### 4.4. RNA Extraction and Reverse-Transcription PCR from Human Tissue

Total tissue RNA was extracted by trizol reagent according to the manufacturer’s protocol (Takara Bio Inc., Otsu, Japan). The extracted RNA with an A260/A280 ratio of 1.9 to 2.1 was considered to be pure and was used in subsequent experiments. Two micrograms RNA was reverse-transcribed into cDNAs using Primescript RT reagent Kit with gDNA Eraser (Takara Bio Inc., Japan). Real-time PCR was performed using the SYBR Premix DimerEraser kit (Takara Bio Inc., Japan). Thermal cycling conditions were as follows: 30 s at 95 °C, then 40 cycles of 5 s at 95 °C, 30 s at 55 °C and 30 s at 72 °C. All the experiments were performed in duplicate. Primers used for real-time PCR are shown in Table 3. The relative expression of target mRNA was normalized to the expression level of GAPDH mRNA using the 2^−∆*C*t^ method.

### 4.5. Statistical Analysis

The SPSS16.0 (SPSS Inc., Chicago, IL, USA) software was used for general statistics analyses. Comparisons between two experimental groups were performed using Student’s *t*-test. Data are expressed as mean ± standard deviation (SD) values. Survival rate was calculated using the Kaplan-Meier method with the log-rank test applied for comparison. All tests performed were two sided and the criterion for statistical significance was *p* < 0.05.

## 5. Conclusions

In conclusion, we identified *PPIC*, *EMP3* and *CHI3L1* as highly discriminative predictors of prognosis. Furthermore, these genes were also found to be highly expressed in glioma tissue compared to normal brain tissue. Furthermore, the prognostic value of the three-gene signature was statistically significant in grades, age and therapy. Therefore, we propose that *PPIC*, *EMP3* and *CHI3L1* may be suitable as prognostic genes or therapeutic targets for high grade gliomas.

## Figures and Tables

**Table 1:** Correlation between PPIC/EMP3/CHI3L1 expression and glioma clinicopathologic features in 70 patients.

	*N*%	PPIC Expression Levels				EMP3 Expression Levels				CHI3L1 Expression Levels			
		High	Low	Ratio	*p*	High	Low	Ratio	*p*	High	Low	Ratio	*p*
		Expression	Expression	(High/Low)		Expression	Expression	(High/Low)		Expression	Expression	(High/Low)	
Sex													
Male	48 (68.57)	13	35	0.371	0.184	16	32	0.5	0.835	15	33	0.454	0.441
Female	22 (31.42)	10	12	0.833		5	17	0.294		7	15	0.467	
Age, year													
<45	45 (64.28)	13	32	0.406	0.068	14	31	0.452	0.762	13	32	0.406	0.965
≥45	25 (35.72)	11	14	0.786		7	18	0.389		9	16	0.563	
Grade													
Low (I + II)	38 (54.28)	7	31	0.226	0.005	11	27	0.407	0.876	13	25	0.52	0.935
High (III + IV)	32 (45.71)	16	16	0.500		10	22	0.454		9	23	0.391	

**Table 2:** Clinical and histological characteristics of patients with glioma.

Sequence	GSE4412	GSE7696	GSE4290	TCGA
Patients (*n*)	74	60	157	567
Male	28	43		347
Female	46	17		220
Age (years)	46 (18–82)	48 (33–70)		58 (10–89)
Grade (*n*)				
I				
II			45	
III	24		31	
IV	50	60	81	567
Temozolomide (TMZ) (*n*)				
Yes				166
No				401
Radiotherapy Z (*n*)				
Yes				422
No				145

**Table 3:** Primer sequences used for real-time PCR.

Gene	Sequence	Base
*PPIC*	F: AGCAAGTTTCATCGTGTCATCA	22
	R: TGGAAATGTCTCACCATAGATGC	23
*EMP3*	F: GGAGGTCTCTTCTATGCCACC	21
	R: AGGATCTCCTCGGCGTGAAT	20
*CHI3L1*	F: GTGAAGGCGTCTCAAACAGG	20
	R: GAAGCGGTCAAGGGCATCT	19
*GAPDH*	F: GAGTCAACGGATTTGGTCGT	20
	R: TTGATTTTGGAGGGATCTCG	20

## Supplementary Materials

Supplementary materials can be found at www.mdpi.com/1422-0067/17/11/1808/s1.
